# Space-time patterns in maternal and mother mortality in a rural South African population with high HIV prevalence (2000–2014): results from a population-based cohort

**DOI:** 10.1186/s12889-017-4463-9

**Published:** 2017-06-03

**Authors:** B. Tlou, B. Sartorius, F. Tanser

**Affiliations:** 1 Discipline of Public Health Medicine, School of Nursing and Public Health, University of KwaZulu-Natal, Durban, South Africa; 2 Africa Health Research Institute University of KwaZulu-Natal, Mtubatuba, South Africa; 3 Centre for the AIDS Programme of Research in South Africa- CAPRISA, University of KwaZulu-Natal, Congella, South Africa

**Keywords:** Maternal mortality, Spatial-temporal clustering, Risk factors, Rural South Africa

## Abstract

**Background:**

International organs such as, the African Union and the South African Government view maternal health as a dominant health prerogative. Even though most countries are making progress, maternal mortality in South Africa (SA) significantly increased between 1990 and 2015, and prevented the country from achieving Millennium Development Goal 5. Elucidating the space-time patterns and risk factors of maternal mortality in a rural South African population could help target limited resources and policy guidelines to high-risk areas for the greatest impact, as more generalized interventions are costly and often less effective.

**Methods:**

Population-based mortality data from 2000 to 2014 for women aged 15–49 years from the Africa Centre Demographic Information System located in the Umkhanyakude district of KwaZulu-Natal Province, South Africa were analysed. Our outcome was classified into two definitions: Maternal mortality; the death of a woman while pregnant or within 42 days of cessation of pregnancy, regardless of the duration and site of the pregnancy, from any cause related to or exacerbated by the pregnancy or its management but not from unexpected or incidental causes; and ‘Mother death’; death of a mother whilst child is less than 5 years of age. Both the Kulldorff and Tango spatial scan statistics for regular and irregular shaped cluster detection respectively were used to identify clusters of maternal mortality events in both space and time.

**Results:**

The overall maternal mortality ratio was 650 per 100,000 live births, and 1204 mothers died while their child was less than or equal to 5 years of age, of a mortality rate of 370 per 100,000 children. Maternal mortality declined over the study period from approximately 600 per 100,000 live births in 2000 to 400 per 100,000 live births in 2014. There was no strong evidence of spatial clustering for maternal mortality in this rural population. However, the study identified a significant spatial cluster of mother deaths in childhood (*p* = 0.022) in a peri-urban community near the national road. Based on our multivariable logistic regression model, HIV positive status (Adjusted odds ratio [aOR] = 2.5, CI 95%: [1.5–4.2]; primary education or less (aOR = 1.97, CI 95%: [1.04–3.74]) and parity (aOR = 1.42, CI 95%: [1.24–1.63]) were significant predictors of maternal mortality.

**Conclusions:**

There has been an overall decrease in maternal and mother death between 2000 and 2014. The identification of a clear cluster of mother deaths shows the possibility of targeting intervention programs in vulnerable communities, as population-wide interventions may be ineffective and too costly to implement.

## Background

Maternal mortality echoes a country’s socioeconomic conditions and aspect of life, as well as the public policies that bolster public health activities [1]. Globally, mother death when children are aged less than 5 is due to selected infectious or non-communicable causes assumed to be enhanced by pregnancy, and the rate is still high in many developing countries [2]. There has however, been a reduction in maternal mortality by 44% in some countries (Mauritius, Cape Verde, Angola, Bangladesh, Brazil, Djibouti, Egypt, Ethiopia etc.) between 1990 and 2015, but this was well short of the target of a 75% reduction indicated in the Millennium Development Goal [3]. The patterns of maternal mortality reveal considerable inequity between and within countries, with 99% of maternal deaths occurring in developing countries and only 1% in developed countries [4]. Given this spatial dimension of health inequalities, it is appropriate to analyze health indicators geographically, and to make use of the approaches afforded by Geographic Information Systems technology and geospatial analysis to facilitate better allocation of limited resources [5]. Remote areas in South Africa are the most affected in terms of overall maternal mortality, but with clear spatial variation within these rural settings [6]. Tuberculosis and malaria are the leading indirect causes of maternal deaths, while hypertensive disorders, sepsis and hemorrhage are the leading direct causes in South Africa [7].

Despite the average global decrease, the maternal mortality ratio (MMR) increased rapidly in sub-Saharan Africa from 1990, this region [8] being regarded by some as the epicentre of the HIV pandemic [9, 10]. However, maternal mortality remains a major problem [11] and the increasing impact of non-communicable diseases is expected to further exacerbate it [10, 12, 13]. Maternal mortality in South Africa significantly increased during 1990–2015 [14], with recent data suggesting no progress towards achieving MDG5- the MMR having increased from 108 per 1000 in 1990 to 138 per 1000 in 2015 [15]. According to a recent South African study, the ‘big five’ maternal causes of death are: non-pregnancy related infections, including HIV, complexities of hypertension, antepartum and postpartum haemorrhage, fresh pregnancy losses pertinent to septic abortions, and pre-existing maternal diseases [7].

The temporal change of maternal mortality and its spatial heterogeneity and hyper endemic HIV in typical rural African settings is still unclear. The few studies that have been done have not adequately applied geospatial analysis to identify areas of high maternal mortality in rural settings and how this may vary at a fine geographic resolution [16]. Authentic knowledge on trends in maternal mortality and what drives these is still largely unknown in rural areas. This effort is exacerbated by significant discrepancies in the direct and indirect causes of maternal mortality by geographic area and time [17].

We explored the spatial patterns and trends of maternal mortality in a demographic surveillance research site, the Africa Centre Demographic Information System, one of the largest and most extensive surveillance sites in Africa. We applied spatial analytical techniques to examine the micro-geographical patterns and clustering of maternal mortality in a high HIV prevalence, rural population. HIV-related and all-cause mortality analysis has exhibited strong spatial clustering trends in this population [18, 19], highlighting the need to investigate spatial clustering of maternal mortality. Similarly, identification of the most important maternal mortality risk factors is essential to develop intervention strategies aimed at preventing pregnancy-related complications.

## Methods

This study uses methodology from previously published work [2, 6, 16] and has been split into three sections namely, study area, mortality data and study population as indicated below.

### Study area

**Fig. 1:**
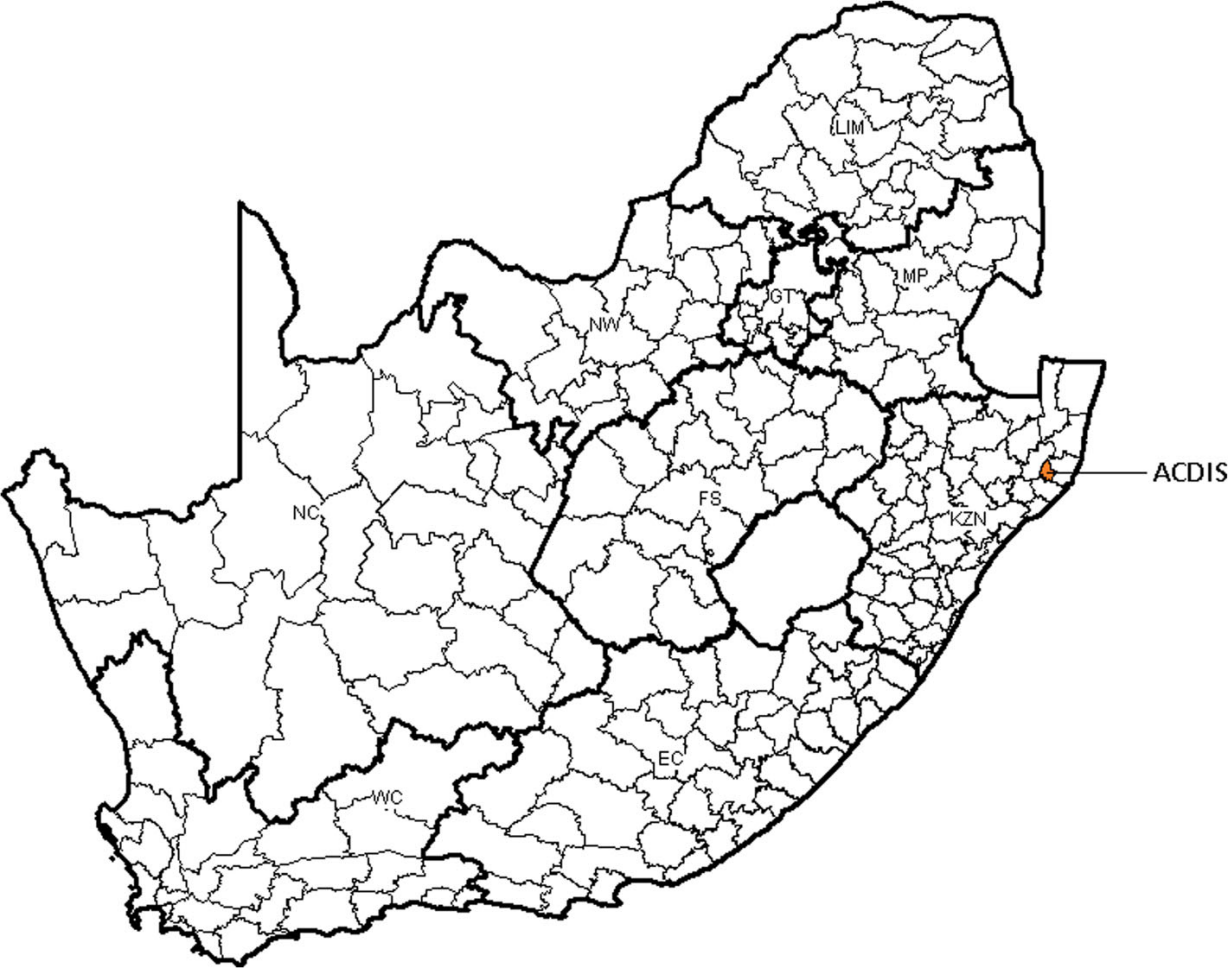
Location of the Africa Centre’s study area in KwaZulu-Natal Province, South Africa

We used longitudinal data obtained from the Africa Centre Demographic Information System (ACDIS) in rural KwaZulu-Natal (KZN) Province, South Africa, which was established in 2000 [20] (Fig. 1). The area is 438 km^2^ in size and includes a population of approximately 90,000 people who are members of approximately 11,000 households. The area is typical of many rural areas of South Africa in that while predominantly rural, it contains an urban township and informal peri-urban settlements. It is characterised by large variations in population densities (20–3000 people/km^2^). In the rural areas homesteads are scattered rather than grouped. Most households are multi- generational and range with an average size of 7.9(SD = 4.7) members [20]. Fieldworkers have diagramed all homesteads and facilities applying differential global position systems [21] to record the geographical locations.

### Mortality data

Qualified medical practitioners ascertained the probable cause of death through interviews carried out with caretakers of the deceased or witnesses of deaths. The method used questionnaires to elicit pertinent information on signs, symptoms, and circumstances leading to death, generically described as indicators, which were subsequently interpreted into causes of death. The medical practitioners recorded a narrative of the circumstances leading up to the death based on the standard INDEPTH/WHO verbal autopsy questionnaire [22, 23].The Africa Centre for Demographic Information System used two methods to determine cause of death for each case: physician coding and an automated method using the InterVA probabilistic verbal autopsy interpretation model, before 1 January 2010 and on or after 1 Jan 2010 respectively [24]. In the physician-coded method, two clinicians independently assigned cause of death on the basis of the information collected during the verbal autopsy and their clinical judgement. If consensus could not be reached between the physicians, a third clinician reviewed all cases and codified the causes of death using the International Classification of Diseases, 10th revision (ICD-10) [25]. The InterVA model is based on Bayesian calculations of probabilities that a particular death was due to particular causes, given a set of symptoms and circumstances associated with the death [26]. The probabilistic model involves the building of a defined set of indicators (signs, symptoms, history, and circumstances) as the components of the model. The model produces the likely causes of death for each case, together with respective likelihoods. A definitive grid of conditional prior probabilities was characterized by a trained panel of physicians [26, 27].

### Study population

We carried out a longitudinal analysis on 38,370 women of child bearing age (15-49 years) registered in the Africa Centre DSA who were born, residing, or in-migrated as a resident in the study area between 2000 and 2014. We determined maternal death using verbal autopsy and defined it, as the death of a woman while pregnant or within 42 days of termination of pregnancy, irrespective of the duration and site of the pregnancy, from any cause related to or aggravated by the pregnancy or its management, but not from accidental or incidental causes. We defined mother death, as the death of the mother while their child was less than or equal to 5 years of age. We included all 1204 deaths (maternal and mother) of women in the age range 15–49 that occurred between 2000 and 2014 in the analysis.

### Statistical analysis

We conducted data analysis using STATA software (version 14) to establish the maternal mortality rates. We calculated the mortality rate using the number of deaths and person-years lived by the women of reproductive age (typically those aged 15 to 49 years) for each year, with a 95% confidence intervals (CI) for mortality rates being computed using the exact CI based on the Poisson distribution.$MMR=\frac{number\  of\  maternal\  deaths}{number\  of\  live\  births}\times 100,000$ Person years.

We then used a logistic regression to identify the risk factors for maternal mortality. We employed the Mosley-Chen and Meade models [28] to identify the risk factors affecting maternal mortality based on three levels: community, household and individual.

### Spatial clustering analysis

We utilized an exponential semivariance model for the spatial kriging of maternal mortality in the ACDIS. For the partial sill and range we assumed the following parameters, namely 10 and 3.3, respectively with no nugget effect. The analysis was performed in R software using the geoR package [29]. To identify circular maternal mortality clustering using spatial and spatial–temporal statistics respectively, we adopted the Kulldorff spatial clustering method [30] using the SaTScan software version 9.3 [31]. The Kulldorff Scan statistic identifies clusters with a higher number of observed cases (maternal deaths) relative to expected cases, under the assumption of spatial randomness, and then evaluates their statistical significance by skimming a circular window that covers the study area. A likelihood ratio test analyses the observed maternal deaths within the circle to the expected maternal deaths across the full range to determine significant risk clusters of mortality, giving relative risk and *p* values for any clusters determined [30]. We ran the model with a maximum cluster size of 50% of the total population, and *p* values achieved across 999 Monte Carlo replications to assure no loss of power at the alpha = 0.05 level [30]. However, one disadvantage of the Kulldorff scan statistic is that it utilises a circular window to denote the possible cluster areas, and cannot detect irregular shaped clusters. We therefore also used, the Flexible spatial scan statistic (Tango spatial scan statistics implemented in FleXScan [32]), in which the identified cluster is both flexible in shape and restricted to relatively small neighbourhoods of each region. The Flexible Scan statistic sets a practicable limitation of a maximum of 30 nearest neighbours for finding possible clusters due to the heavy computational load [32]. We aggregated the data into 705 grid cell size for FlexScan, given its current limitations of being able to search a maximum of 30 adjacent nodes. To refine the extents of the identified space-time cluster using FleXScan, we then used the Kulldorff space-time statistics for cluster detection of maternal mortality.

## Results

### Study population and mortality

**Fig. 2:**
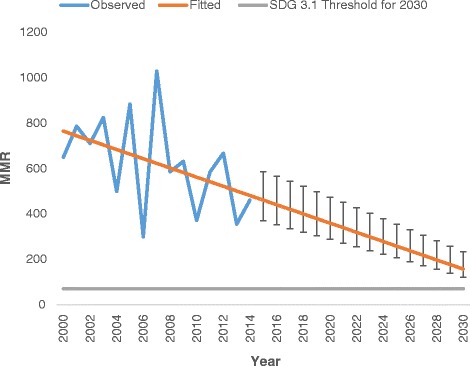
Maternal mortality temporal trends 2000–2014, plus projection to 2030 using non-linear Poisson regression model plus 95% uncertainty intervals (*horizontal grey line* is SDG3.1 target for MMR by 2030 i.e. MMR < 70 per 100,000 live births)

During the 15 years, there were 212 (0.55%) maternal deaths from 32,620 live births, with the maternal mortality ratio being 650 per 100,000 live births. Overall, 1204 mothers died while their child was less than or equal to 5 years giving a mortality rate of 370 per 100,000 live births. The trends for maternal mortality are shown in Fig. 2. The trend line in Fig. 2 reveals some evidence for a maternal mortality rate decline over the study period, from approximately 800 to 400 per 100,000 live births. The linear trend above is statistically significant at the *p* = 0.1 level (IRR = 0.969 [95% CI: 0. 938–1.002], *p*-value = 0.069). If the current rate of decline is maintained, the Sustainable Development Goal (SDG) 3.1 target for MMR by 2030 will be reached in 2024/2025.

### Spatial clustering of maternal mortality

**Fig. 3:**
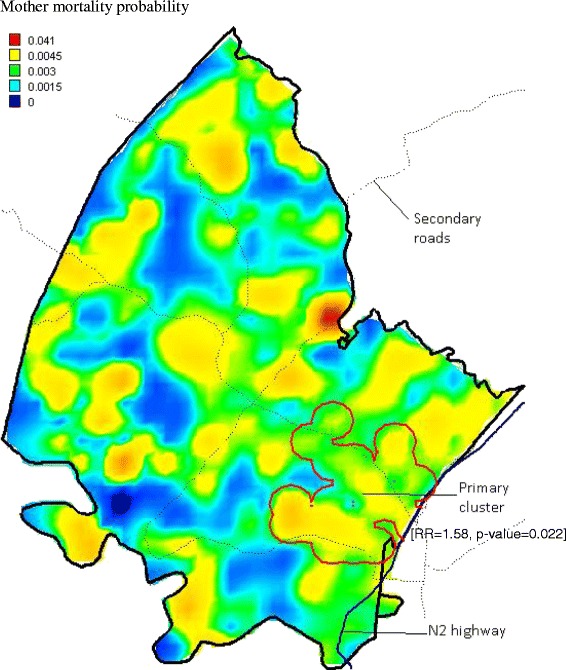
Spatial clustering (based on Tango spatial scan statistic) of mother death (plus kriged probability quintiles) in the DSA, 2000–2014. The relative risk of the primary cluster is 1.58 (*p* = 0.022)

**Fig. 4:**
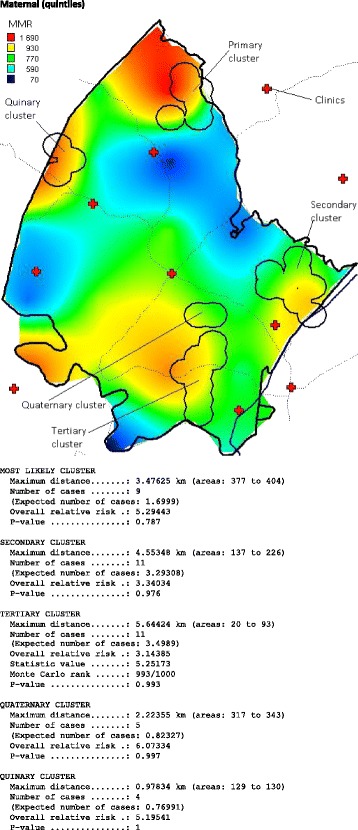
Spatial clustering (based on Tango spatial scan statistic) of maternal mortality (plus kriged probability quintiles) in the DSA, 2000–2014. The relative risk of the primary cluster is 5.29 (*p* = 0.787)

We detected a significant primary cluster of mothers who died when their children were less than 5 years in the south east of the study area, with a relative risk of 1.58 (*p* = 0.022) (Fig. 3). This cluster is a high density peri-urban area located at the intersection of two major roads. Our study found no clear evidence of spatial clustering of maternal mortality events in the study area (Fig. 4). The summarised location, observed maternal deaths, expected maternal deaths, relative risk per each identified cluster are in Table 1.

**Table 1:** Clusters of spatial maternal mortality using the spatial analysis scanning for high mortality rates, DSA, 2000–2014

Characteristic	Location within site	Crude rate per 100,000 live births	Observed cases	Expected cases	Relative risk (RR)	*p*-Value
Maternal mortality	Semi-Urban	650	25	8.4	2.97	0.276
Mother death in childhood	Semi-Urban	370	169	109	1.58	0.022*

*signifies a statistical significant *p*-value

### Risk factors of maternal mortality

We used the Mosley-Chen and Meade models [28] to identify the risk factors affecting child and maternal mortality based on three levels of analysis: community (food distribution, physical infrastructure like railroad, quality of water, electricity, water supply, road networks and political institutions), household (capital, wealth effects (food production, clothing essentials, housing conditions, energy availability, transportation, means to purchase what is necessary for hygienic purposes/preventive care, access to information), and individual (skills, health and time, normally measured by mother’s educational level, whilst father’s education correlates with occupation and household income). Table 2 depicts the baseline characteristics the identified risk factors of maternal mortality.

**Table 2:** Baseline characteristics for risk factors of maternal mortality

	Maternal death	
HIV Positive	Yes	No
Yes	35 (16.5)	2520 (6.6)
No	177 (83.5)	35,638 (93.4)
Highest education level		
Primary or less	87 (88.8)	9938 (78.9)
Secondary or more	11 (11.2)	2659 (21.1)
Age		
15–19	20 (9.4)	6563 (17.2)
20–24	43 (20.3)	7841 (20.5)
25–29	63 (29.7)	7475 (19.6)
30–34	48 (22.6)	6041 (15.8)
35–39	22 (11.8)	4504 (11.8)
40–44	7 (3.3)	3327 (8.7)
45–49	9 (4.2)	2407 (6.3)
Distance to nearest clinic(km)		
**≥** 10 km	3 (2.2)	246 (1.5)
< 10 km	131 (97.8)	15,688 (98.5)
Year		
2000–2005	105 (49.5)	18,832 (49.4
2006–2014	107 (50.5)	19,321 (50.6)

We applied univariable logistic regression to analyze the relationship between maternal death and risk factors, with crude odds ratios and 95% confidence intervals being estimated for each parameter. We then used multivariable logistic regression to build an overall model from the factors that were significantly associated with maternal mortality in the univariable analysis. In the final model, significant associations with the risk of maternal death were age, HIV status, education and parity (Table 3). Primary education or none, being HIV positive, and higher parity were significant predictors of increased risk of maternal death. We also presented the multivariable logistic regression results for factors associated with mother death clusters (Table 4).

**Table 3:** Univariable and multivariable odds ratios (95% CI) for risk factors associated with maternal mortality, 2000–2014

		Univariable			Multivariable		
Explanatory variables	Categories of explanatory variables	Crude odds ratio	Confidence interval	*p*- value	Adjusted odds ratio	Confidence interval	*p*-value
HIV Positive	Yes	2.797	1.94–4.03	<.0001	2.541	1.536–4.202	<.0001
	No	1			1		
Highest education level	Primary or less	2.116	1.13–3.97	0.019	1.972	1.040–3.740	0.038
	Secondary or more	1			1		
Age (years)	15–19	1			1		
	20–24	1.80	1.06–3.06	0.03	1.704	0.800–3.629	0.167
	25–29	2.766	1.67–4.58	<.0001	1.785	0.857–3.720	0.122
	30–34	2.607	1.55–4.40	<.0001	1.058	0.471–3.475	0.892
	35–39	1.603	0.87–2.94	0.127	0.585	0.224–1.524	0.272
	40–44	0.690	0.29–1.63	0.399	0.366	0.105–1.272	0.114
	45–49	1.227	0.56–2.70	0.611	0.130	0.015–1.107	0.062
Parity		1.268	1.20–1.34	<.0001	1.422	1.243–1.627	<.0001
Distance to nearest clinic	≥10 km	1.46	0.46–4.62	0.519			
	<10 km	1					
Household electrified	No	1.48	0.88–2.51	0.142			
	Yes	1					
Year	2000–2005	1.007	0.77–1.32	0.961			
	2006–2014	1

**Table 4:** Univariable and multivariable odds ratios (95% CI) for factors associated with mother death clusters, 2000–2014

		Univariable			Multivariable		
Explanatory variables	Categories of explanatory variables	Crude Odds ratio	Confidence interval	*p*- value	Adjusted odds ratio	Confidence interval	*p*-value
Death due to AIDS/TB	Yes	2.722	2.31–3.21	<.0001	2.183	1.667–2.857	<.0001
	No	1			1		
Highest education level	Primary or less	2.504	1.68–3.73	0.019	2.122	1.404–3.207	<.0001
	Secondary or more	1			1		
Age (years)	15–19	1			1		
	20–24	0.892	0.69–1.16	0.388	0.764	0.502–1.165	0.211
	25–29	0.864	0.66–1.12	0.275	0.661	0.434–1.006	0.533
	30–34	1.014	0.78–1.33	0.916	0.685	0.444–1.057	0.087
	35–39	1.017	0.76–1.36	0.908	0.532	0.324–0.871	0.012
	40–44	1.103	0.81–1.51	0.538	0.879	0.531–1.455	0.615
	45–49	1.834	1.37–2.47	<.0001	1.262	0.736–2.165	0.397
Parity		1.137	1.09–1.19	<.0001	1.086	0.998–1.181	0.057
Distance to nearest clinic	≥10 km	1.384	0.80–2.39	0.243			
	<10 km	1					
Household electrified	No	1.285	0.99–1.68	0.163			
	Yes	1					
Year	2000–2006	1.495	1.27–1.76	<.0001	1.197	0.928–1.544	0.165
	2007–2014	1			1		
Socio-economic status	Poor	1.125	0.71–1.79	0.300			
	medium	0.698	0.35–1.38	0.619			
	Rich	1					

## Discussion

Our results suggest that there has been a declining trend in maternal mortality from 2000 to 2014 in this rural population, in line with the global trend. This could be attributed to various interventions at both national and district levels, such as antiretroviral therapy introduced in 2004, community health funds for better health care, and improvements in antenatal services, obstetric care and food security in South Africa. The spatial pattern showed marked geographic differences in maternal mortality indicating that maternal mortality was not evenly distributed across the DSA. We found that, parity, HIV status, education and age were significant predictors of maternal mortality.

The results of our investigation are in line with the findings of a maternal mortality study for 181 countries done in 2015 [11]. The previous work showed that, global maternal mortality declined from 390,185 (95% UI 365193–416,235) in 1990 to 374,321 (351336–400,419) in 2000 before dropping to 275,288 (243757–315,490) in 2015. The whole reduction from 1990 to 2015 in universal maternal deaths was approximately 29% and the decline in MMR was 30%. Similarly, MRR followed the same trend to overall maternal deaths; MMR was 282 (95% UI 264–300) in 1990, 288 (270–308) in 2000, and fell to 196 (173–224) in 2015. The study also showed geographical differences in maternal mortality [11].

Other research done in rural KwaZulu - Natal (Amajuba district) showed declining trends of MMR post 2006 because of ART rollout [33]. Recent data from World Health Organization (WHO) suggests that there has been limited or no development in South Africa regarding maternal mortality, as it has increased from 108 per 1000 in 1990 to 138 in 2015, largely due to HIV/AIDS [15]. In 2011, Dorrington and Bradshaw did an investigation on maternal mortality from many different sources in South Africa, covering national census reports and household reviews, assessing the disparities between them regarding definitions, data and methodological weaknesses. They found discrepancies in maternal mortality estimates due to variations and inaccuracies in data processing [34].

The predicament of measuring the evolution on maternal deaths in South Africa is that there are no steady population-based estimates that conclusively depict the trends of MMR [35].

The Africa Centre DSA data comprises of all maternal deaths that took place in the study area population, irrespective of the place of death over the study period. This involves deaths that transpired at home, in hospitals, en route for care, or somewhere, and thus the rates attained from the DSA vary from those attained by other methods, particularly from the classified inquisitions. DSA estimates also vary from the vital registration projections, particularly for pregnancy-linked deaths, largely because the data for pregnancy is concluded in only a minor percentage of deaths of women aged 15–49 years [36]. Enhancing the scope of the vital registration system, as well as enhancing the entry of system forms, is decisive for thoroughly supervising the accelerated dynamic levels of maternal mortality. The authentic measurement of maternal mortality is a challenging undertaking, which involves the detailed recording of deaths and their roots. In the lack of substantial vital registration systems, health service records, household surveys and census data are used as alternatives to estimate maternal mortality [37].

In addition, maternal mortality patterns emphasize the huge variations triggered by emerging infectious diseases in South Africa, a country experiencing swift and complicated health transitions. These fluctuations may be a direct result of the evolution of the HIV/AIDS epidemic, and past investigations conducted to assess risk factors for maternal deaths in ACDIS have shown that, HIV status and parity are linked with increased risk of maternal death [38]. Also, previous studies in Sub-Saharan Africa have identified education [39], HIV and parity [40, 41] as prominent risk factors for maternal mortality. The findings of this study highlights the need to integrate spatial disparities of maternal mortality, and the estimation of risk factors as well as exploring the limitations of the prevailing health information systems in South Africa.

While the national target for MDG 5 was not achieved, there is some indication of a decrease in maternal mortality in this typical rural population. The recent proof suggests that there is a greater logic for optimism than has been thought, and that significant reductions in the MMR are feasible over a brief period. In addition, there is now a recognition that the ART programme has decreased maternal mortality, as HIV infection has been attributed to the large increase in maternal mortality. Thus, maternal mortality, in the field of public health and reproductive rights, is very important and should be treated as a fact that can be avoided by health professionals.

The main strength of the study is the large population under surveillance in the Africa Centre demographic information system and the rigorous demographic surveillance system which continuously captured vital population statistics (births, deaths and migration) longitudinally. This provided a platform for a reliable person-time of exposure which enabled the calculation of accurate maternal mortality rates which were free from the influence of stillbirth prevalence and induced abortion that are present in the maternal mortality ratio calculation. More still, the use of a population-based sample in the study limited the issue of selection bias that would otherwise be introduced by hospital-based studies and results obtained are consistent and comparable with other research findings in other settings; hence, the authenticity of the results was not compromised**.** The limitations of the study includes the VA method [20] used to ascertain the probable cause of death of the study participants using information on symptoms and signs gathered during bereavement interviews of persons who were caring for the deceased. This is not ideal as there are issues regarding validity in assessing the cause of death due to recall bias, response errors or misclassification of mortality during the coding process. However, this is not an issue in this study as qualified medical practitioners ascertained the cause of death based on the symptoms of the deceased.

## Conclusions

We have demonstrated clear evidence of spatial clustering in mothers who died when their children were less than 5 across the study area. Since understanding spatial patterns of a health-related problem is one of the basic tenets of public health [42], these results provide a rationale for the need to target intervention programmes to areas where mother death is most likely to occur. Population-wide interventions may be too costly to implement and ineffective to markedly decrease in maternal mortality and studies have shown that community-focused interventions in similar settings successfully bring about reduction in mother mortality [43, 44].

## Abbreviations

ACDIS: Africa centre demographic information system
CI: Confidence intervals
DSA: Demographic surveillance area
KZN: KwaZulu-Natal
VA: Verbal autopsy
WHO: World Health Organisation
